# Melatonin and sleep parameters in infertile women with endometriosis: first results from the triple-blind randomized controlled trial of administration of melatonin in chronic pelvic pain and sleep disturbance

**DOI:** 10.1371/journal.pone.0321635

**Published:** 2025-04-16

**Authors:** Sedighe Esmaeilzadeh, Fatemeh Habibolahi, David Moher, Zahra Basirat, Hemat Gholinia, Masoumeh Golsorkhtabaramiri, Parvaneh Mirabi

**Affiliations:** 1 Infertility and Reproductive Health Research Center, Health Research Institute, Babol University of Medical Sciences, Babol, Iran; 2 Student Research Committee, Babol University of Medical Sciences, Babol, Iran; 3 School of Epidemiology and Public Health, University of Ottawa, Ottawa, Canada; University of Milan, ITALY

## Abstract

**Introduction:**

To evaluate the effect of melatonin supplementation on sleep quality and pelvic pain in infertile women with endometriosis and sleep disturbances.

**Materials and analysis:**

A randomized, triple-blind, placebo-controlled trial was conducted among 80 infertile women with endometriosis and sleep disturbances. Participants were randomly assigned to receive either 5 mg melatonin or placebo for 2 months. The primary outcome was change in overall sleep quality as measured by the Pittsburgh Sleep Quality Index (PSQI). Secondary outcomes included changes in specific PSQI domains and chronic pelvic pain.

**Result:**

Among 80 infertile women with endometriosis and sleep disturbances, melatonin significantly improved overall sleep quality compared to placebo, with a large effect size (p < 0.001, η² = 0.20, Cohen’s d=1). The mean difference in sleep quality was a reduction of -1.7 on the PSQI, although it did not reach the clinically meaningful threshold of 3 points. Melatonin also led to considerable improvements in specific PSQI domains including: a substantial increase in sleep duration and a marked reduction in sleep disturbances. Furthermore, melatonin significantly decreased sleep latency, exhibiting a large effect size, and contributed to a medium reduction in the use of sleep medications. However, no significant improvements were noted in sleep efficiency, daytime dysfunction, or subjective sleep quality. Additionally, melatonin significantly reduced chronic pelvic pain, with a large effect size (p < 0.001, η² = 0.18, Cohen’s d = 0.93).

**Conclusion:**

While melatonin may improve sleep quality and reduce pelvic pain, further investigation is needed to assess its clinical relevance in this population.

**Trial registration:**

ClinicalTrials.gov IRCT20171209037794N4.

## Introduction

Endometriosis-associated chronic pelvic pain (EACPP) is a condition that can significantly impair sleep quality [1]. The relationship between pain and sleep is a mutual cycle, where pain leads to sleep deprivation and poor sleep increases pain sensitivity. Consequently, sleep disturbances can exacerbate depressive thoughts and reduce functional quality of life in individuals with endometriosis [2–4]. Few studies have shown higher scores on the Insomnia Severity Index and Epworth Sleepiness Scale among endometriosis patients. Sleep disturbances in this population can be understood as a complex interplay of factors, including pain, hormonal changes, and psychological stress, but the specific focus on sleep-related issues in these patients has yet to receive adequate attention in the literature [5,6].

Sleep disturbances significantly affect physical and mental health. Poor sleep quality, characterized by increased latency and disturbances, is linked to higher fatigue levels and can lead to chronic pain and mood disorders like anxiety and depression. Given the connection between disrupted sleep and chronic pain conditions, it is reasonable to conclude that these disturbances may severely impact the quality of life for individuals with endometriosis [7].

Many women seeking relief from their symptoms turn to natural alternatives due to concerns about side effects, dependence, and diminishing effectiveness of long-term pharmacologic treatments. Natural remedies often provide a holistic approach with fewer adverse effects, but it’s important for women to consult healthcare professionals before starting any new treatment. Melatonin is a hormone produced primarily by the pineal gland and can regulate circadian rhythms. It manages endometriosis by regulating endometrial epithelial cell function [8]. In a controlled trial study, the melatonin- treated group showed fewer clinical biomarker of endometriosis (brain-derived neurotropic factor (BDNF) compared to that of healthy individuals [9]. Thus, it contributes to the improvement of EACPP, leading to a higher quality of life. Antinociceptive antioxidant and anti-inflammatory effects of melatonin has been stated in some animal models of acute and inflammatory pain [10], also in the few human studies, doses of 3–10 mg of melatonin was more effective than a placebo in improving sleep quality among endometriosis patients with EACPP [11].

While primarily recognized as a sleep aid, melatonin also offers various benefits, including the potential to lower BDNF levels, which can significantly alleviate all types of pain, such as inflammatory, postoperative, and experimental pain [12]. Sleep deficiency is associated with chronic pain, ranging from 40% to 50% of patients with pain Obviously, pain relief is a major goal, but the additional treatment of sleep disturbances may lead to a further decrease in pain [13]. Painful stimuli affect salivary melatonin levels. Decrease in endogenous melatonin exacerbates neuropathic pain caused by nerve injury, whereas administration of melatonin significantly reduces neuropathic pain caused by sleep deprivation [14]. However, another study revealed that melatonin’s effect on pain is independent of improvements in sleep quality [15].

Physicians often prescribe chemical analgesics to alleviate symptoms of endometriosis and improve sleep quality; however, many women with EACPP seek natural alternatives. Unfortunately, sleep disorders in this population have received limited attention, and relevant results remain unpublished. In our previous study, currently under review, we found that melatonin significantly reduced dysmenorrhea in this population. Building on these findings, the present study aims to investigate the effects of melatonin on sleep disturbances associated with chronic pelvic pain, thereby elucidating its potential therapeutic benefits for infertile women affected by endometriosis.

## Methods and analysis

### Study design

This is a single center; triple blind, parallel group randomized controlled trial. The study protocol was prospectively registered in the website of Iranian Registry of Clinical Trials as IRCT20171209037794N4. The study protocol is based on the Standard Protocol Items: Recommendations for Interventional Trials 2013 guideline.

### Ethics approval and consent to participate

This research was approved by Institutional Review Board and Institution Ethics Committee at Babol University of Medical Sciences (No.MUBABOL.HRI.REC.1402.041), Dated 03/07/2023.The consent form of our patients was taken.

### Study setting

This trial was performed at the Fatemezahra Infertility Center, a referral center for infertility problems in northern Iran that is affiliated with Babol University of Medical Sciences. This center is one of the best-equipped therapeutic infertility centers in the region, specializing in the diagnosis and treatment of various infertility-related conditions, including endometriosis.

### Participants

Eighty infertile patients diagnosed with endometriosis and sleep disorders were recruited for this trial after providing informed consent and meeting the eligibility criteria. Forty patients in the experimental group received melatonin (5 mg), while 40 participants in the comparison group received a placebo. The diagnosis of endometriosis was based on laparoscopy, and its staging was determined according to the consensus of the Global Endometriosis Society. Patients with EACPP completed the Pittsburgh Sleep Quality Index (PSQI) questionnaire to evaluate sleep quality.

Eighty infertile patients diagnosed with endometriosis and sleep disorders were recruited for this trial after providing informed consent and meeting the eligibility criteria. The diagnosis of endometriosis was confirmed by laparoscopy, and its staging was determined according to the consensus of the Global Endometriosis Society. Patients with endometriosis-associated chronic pelvic pain (EACPP) were evaluated using the Pittsburgh Sleep Quality Index (PSQI) questionnaire to assess their sleep quality.

### Eligibility criteria

The eligibility of each included patients was confirmed by the gynecologist. Subjects eligible for the study had to meet all of the following criteria at randomization:

- 18–45 years of age, who had complained of endometriosis with EACPP (VAS≥4), and Sleep disturbances based on PSQI questionnaire (Score ≥ 5).
- No history of surgical or medical treatments for infertility.
- Not smoking, drinking alcohol, and addiction to substance.
- Lack of systemic illness diseases, such as diabetes, blood pressure, seizures.

### Exclusion criteria

- Continuous use of sleeping pills, anticoagulants and anticonvulsants
- Women who were treated with antidepressants
- Use of stimulants or hypnotics or anti-anxiety medications
- Recent diagnosis of mood disorders or neuropsychiatric symptoms (within 8 weeks)
- Pregnancy or breastfeeding
- Known hypersensitivity to melatonin
- If the patient did not want to continue to participate
- Ongoing or previous pharmacologically treated depression or bipolar disorder

### Work involving nightshifts

#### Intervention.

Patients were assigned in a 1:1 ratio to receive either melatonin or placebo at the same time every night, approximately 2 hours before regular bedtime. In the melatonin treatment group, patients received a 5 mg melatonin tablet once a day for 2 months. The melatonin was a prolonged-release formula (Jalinous Company, Melatonin 5 mg). The comparison group received a daily placebo treatment that was identical in appearance and consisted of starch (Jalinous Company). All patients were allowed to use analgesics (acetaminophen, ibuprofen, codeine) as needed to relieve pain. However, before taking any analgesics, it was emphasized that the maximum intensity of pain should be measured and noted in the questionnaire before using their usual medication. The number and types of analgesics used before and during the treatment were recorded by participants and monitored monthly by the investigator. If pain persisted, patients could take acetaminophen and codeine. Patients were asked to record the type and amount of analgesic used during treatment.

Demographic information, clinical information, ultrasound reports, laparoscopy findings, and the PSQI questionnaire were completed prior to the intervention. After the procedure, the questionnaire was given to the participants along with the medication package. The participants were asked to complete the intervention over a period of 2 months.

#### Follow-up.

One month after the intervention, participants were monitored at the endometriosis clinic, and a checklist of their pain scores and possible side effects was completed. To prevent attrition and assess adherence to treatment, telephone interviews were conducted every 21 days. Two months after the intervention, all participants were examined at the fertility clinic. The self-perceived PSQI questionnaire and pain VAS score was completed again by a midwife not involved in the trial.

#### Outcome measurement.

The primary outcome measure was the change in sleep quality after two months in patients who received melatonin compared to women who received placebo, using the PSQI questionnaire. The PSQI consisted of 19 questions to evaluate sleep quality in the previous month. The global PSQI score reflected the sum of the seven components (sleep duration, sleep disturbance, sleep latency, sleep efficiency, daytime dysfunction, and sleep quality) and ranged from 0 to 21. Higher scores indicate poorer sleep quality, with a score greater than 5 suggesting significant sleep difficulties.

Pelvic pain was also assessed as a secondary endpoint of the present trial using the VAS score. The mean value of the difference between baseline and 2 months later was compared between the two groups. A self-assessment patient checklist was used to evaluate adherence to treatment. Reasons for dropping out and non-compliance were also noted and reported.

#### Sample size.

PSQI scores were presented as the primary outcome. The sample size was calculated based on the minimal clinically important difference (MCID) approach for sleep quality as measured by global PSQI scores for the primary outcome. In the previous study we assessed the effect of melatonin on dysmenorrhea and chronic pelvic pain, by using Biberoglu and Behrman scale among 98 endometriosis patients (Esmaeilzadeh et al, 2024, under review).

In the current trial, we assumed means of 4.91 in the melatonin group and 7.91 in the placebo group [16], assuming a pooled standard deviation of 3.71 and an effect size (Cohen’s f) of 0.404 in a two-tailed test with α=0.05α=0.05 and power of 0.9; The trial was powered to detect MCID of 3 points (on the PSQI) between the 2 groups at the end of the study. Allowing for a 20% drop-out rate, a sample size of 40 participants for each group was needed.

### Recruitment

The study was conducted at the Fatemezahra Infertility Center, in northern Iran, which is affiliated with the Babol University of Medical Sciences. During the run-in phase of the study, a dedicated research team comprising a senior investigator, a medical student, and a gynecologist were present at the endometriosis clinic of the Infertility Center four days a week.

This research team played a crucial role in the recruitment process. They actively engaged with potential participants, informed them about the relevant aspects of the study, and screened all subjects for eligibility criteria at the time of enrollment. The screening process involved assessing the participants’ sleep quality using the Pittsburgh Sleep Quality Index (PSQI) and evaluating their chronic pelvic pain intensity with the visual analog scale (VAS). To be eligible for the study, participants were required to have a PSQI global score of ≥5, indicating the presence of identifiable sleep disturbances, as well as a confirmed diagnosis of chronic pelvic pain with a VAS score of ≥4. The screening and enrollment of participants continued until the target sample size was achieved.

The recruitment phase for this study commenced on July 22, 2023, and was completed on February 2, 2024, over a period of approximately 6 months. This extended recruitment period ensured that the desired sample size could be reached, as the study aimed to enroll a total of 80 participants (40 in each treatment group).

The recruitment phase for this study commenced on July 22, 2023, and was completed on February 2, 2024, over a period of approximately 6 months.

### Pre-randomization data

Demographic characteristics and a complete medical history were obtained at the initial visit, along with information through a clinical interview that included age, occupation, body mass index, pelvic pain intensity, medical and drug history, endometriosis pelvic ultrasound findings, and endometriosis laparoscopy findings. After a gynecologist determined that a patient was eligible for recruitment, all eligible participants were asked to sign a written informed consent form prior to enrollment in the study. The PSQI was completed at the start of the baseline intervention and again after 2 months of treatment.

### Allocation (method & allocation concealment mechanism)

Permuted block randomization was used to achieve balance in the allocation of participants to intervention arms. Before the initiation of the run-in phase of the study, two sets of 40 random numbers were created by a member of the research team who was not involved in recruitment. Twenty blocks of size 4, with a combination of A and B, were prepared. Randomization was carried out using dedicated online software (http://www.randomization.com). The allocation sequence was password-protected and accessible only to the independent investigator who was not involved in the study.

### Blinding

The administration of medication type was triple-blinded. All parties were blinded during the random allocation sequence to avoid selection bias. Blinding of medication conditions was conducted by a pharmacist at Shahid Beheshti University of Medical Sciences. A placebo tablet, identical in size and appearance to the melatonin tablet (Jalinous Pharmaceutical Company), was given to the placebo group.

### Data collection

After checking the baseline characteristic data and eligibility criteria, the PSQI questionnaire was completed at baseline and 2 months after enrollment. Chronic pelvic pain was defined as the presence of intense non-cyclic pain in the pelvic area lasting for at least six months and was measured using a 10-cm VAS. Based on previous studies [17] and opinions of neurologist we considered a change in sleep quality of more than 3 points on the PSQI as the minimal clinically important difference.

### Data monitoring

A faculty member from BUMS (Dr. Alijan Ahmadi Ahangar, Professor of Neurology), who was not involved in the trial and was chosen by the vice chancellor of the university, monitored the data and supervised the conduct of the study. Participants were prescribed medication during the first visit when recruitment for the study occurred. Three weeks after the treatment, a telephone interview was conducted to clarify side effects and to remind participants about their medications. If participants withdrew for any reason, they were asked to complete an early termination assessment. They were expected to be seen in the clinic as soon as possible following discontinuation of the study drug.

### Statistical analysis of baseline characteristics and outcomes

Statistical analysis was conducted using the Statistics Package for Social Sciences software (version22, SPSS, Chicago, IL, USA) and Stata 14 (Stata Corp, College Station, TX, USA), following the intention-to-treat (ITT) approach. All analyses were performed with two-sided uncertainty, and a significance threshold was set at a type I error rate of 0.05. Baseline characteristics of participants were compared between the melatonin and placebo groups using independent t-tests for normally distributed continuous variables and Chi-squared tests for categorical variables. To evaluate between-group differences over time—from pre-intervention to post-intervention—the mean difference in PSQI scores between the melatonin and placebo groups was analyzed using analysis of covariance (ANCOVA). The ANCOVA models included the “After Intervention” values of the outcome variables (e.g., overall PSQI score, sleep duration, sleep disturbance, sleep latency, sleep efficiency, daytime dysfunction, and overall sleep quality) as the dependent variables. The treatment group (melatonin vs. placebo) was included as the independent variable, and the corresponding “Before Intervention” values of the outcome variables were entered as covariates. This approach allowed us to adjust for any baseline differences between the melatonin and placebo groups on the outcome measures, thereby enhancing the interpretation of the treatment effects. The impact of the intervention was assessed through the ANCOVA analyses, and effect sizes were estimated using eta-squared (η²) to provide an estimate of the magnitude of the treatment effects. The interpretation of partial eta squared values is as follows: a small effect size is 0.01, a medium effect size is 0.06, and a large effect size is 0.14 or higher. Additionally, Cohen’s d was calculated to facilitate the interpretation of the results, with thresholds of 0.2,0.5, and 0.8 denoting small, medium, and large effect sizes, respectively (Cohen,1988). The trial is reported using the CONSORT 2010 reporting guideline.

## Results

### Participant characteristics

**Fig 1:**
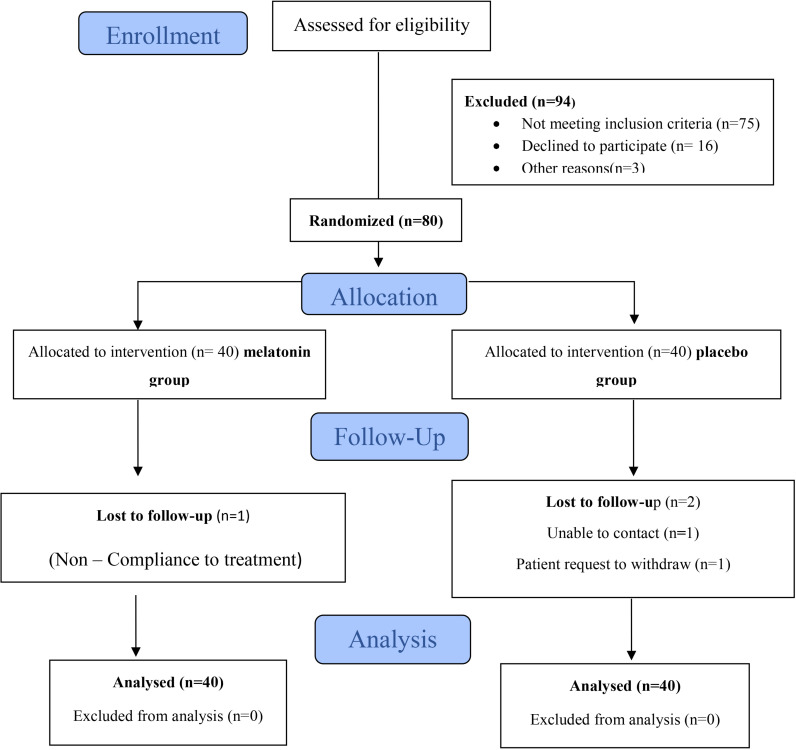
Flow of participants through the study.

In this trial, the effect of melatonin on sleep quality was evaluated in infertile women diagnosed with endometriosis and experiencing sleep disturbances. One hundred seventy-four eligible infertile women with laparoscopy confirmed endometriosis were screened and 80 women were enrolled in accordance to the eligibility criteria. Of the total of 80 participants, 3 of them withdrew during follow-up. Reasons for lost to follow-up are shown in Fig 1.

Table 1 presents the demographic characteristics of both groups, showing comparable distributions of age, body mass index (BMI), type of infertility, and endometriosis staging. This random allocation effectively balanced the groups regarding confounding variables, thereby enhancing the reliability of the statistical analyses conducted. The ANCOVA results indicated that the melatonin group exhibited significantly improved overall sleep quality compared to the placebo group. The mean difference in overall sleep quality between the melatonin and placebo groups was -1.7 (95% CI: -2.63 to -0.77), indicating a statistically significant improvement in the melatonin group This finding was accompanied by a large effect size, as evidenced by an eta squared of 0.20 and a Cohen’s d=1, However, the study was powered to detect a minimal clinically important difference of 3 points between groups, and the observed mean difference of -1.7 may not reach clinical significance despite being statistically significant. Adjusting for age did not alter the significance of the results, with a p-value less than 0.001 and a Cohen’s d of 1.06.

**Table 1:** Demographic Characteristics of Participants.

Variable	Melatonin n=40	Placebo n=40
**Age (Mean ± SD)**	32.6 ± 5.74	31.8 ± 4.77
**BMI (Mean ± SD)**	25.38 ± 3.73	25.67 ± 3.70
**Duration of infertility**	4.46 ± 3.35	4 ± 2.48
**Staging of endometriosis**		
Stage I, II	12 (30)	13 (32.5)
Stage III	14 (35)	10 (25)
Stage IV	14 (35)	17 (42.5)
**Type of Infertility**		
Primary	27 (67.5)	26 (65)
Secondary	13 (32.5)	14 (35

Data presented as Mean (SD) and N (%) for continuous and categorical variables, respectively

Melatonin supplementation also had a considerable effect on specific PSQI domains. The intervention group showed a medium increase in sleep duration and a large reduction in sleep disturbances (Table 2) Additionally, melatonin significantly decreased sleep latency, demonstrating a large effect (Cohen’s d= 0.80), and a medium reduction in sleep medications (Cohen’s d= 0.58). However, no significant improvements were observed for sleep efficiency, daytime dysfunction, or subjective sleep quality (Table 2). Regarding pelvic pain, melatonin had a significant effect (p <0.001), with a mean difference of 1.56 (95% CI: 0.71, 2.41) and a large effect size (Cohen’s d =-0.93). Despite achieving statistical significance, the observed mean difference of 1.56 may not meet the threshold for clinical significance, as our a priori hypothesis anticipated a reduction of at least 1.8 units on the Visual Analog (Table 2). Adjusting for age did not alter the significance of the results, with p-values remaining less than 0.001. This robustness was accompanied by a large effect size, as indicated by an eta squared of 0.17 and a Cohen’s d of 0.90. These findings suggest that the intervention’s effects on the outcomes were substantial and consistent across different age groups.

**Table 2:** Changes in Sleep Quality Components and Pelvic Pain over Time in melatonin and Control Groups.

Variables	Group^*^	Before Intervention (Mean ± SD)	After Intervention (Mean ± SD)	P-value^**^	Difference in change	95% Confidence Interval	Eta Square
**Overall PSQI Score**	Melatonin	9.05 ± 2.50	6.90 ± 1.93	**p < 0.001** ^**$**^	-1.7	-2.63, -0.77	0.20
	Placebo	8.65 ± 2.13	8.20 ± 1.77				
**Sleep Duration**	Melatonin	2.25 ± 0.54	1.90 ± 0.70	p = 0.01	-0.08	-0.40, 0.25	0.07
	Placebo	1.95± 0.74	1.98 ± 0.76				
**Sleep Disturbance**	Melatonin	7.77 ± 3.2	5 ± 2.20	**p < 0.001**	-2.22	-3.26, -1.18	0.28
	Placebo	7.75 ± 2.25	7.22 ± 2.44				
**Sleep Latency**	Melatonin	0.88 ± 0.75	0.40 ± 0.59	**p < 0.001**	0.38	0.08, 0.66	0.14
	Placebo	0.83 ± 0.67	0.78 ± 0.69				
**Sleep Efficiency**	Melatonin	84.83 ± 18.85	88.68 ± 17.95	p = 0.90	-3.26	-9.89, 2.97	0.00
	Placebo	91.05 ± 11.56	91.94 ± 9.81				
**Daytime Dysfunction**	Melatonin	1.23 ± 0.83	1.13 ± 0.75	p = 0.24	-0.17	-0.51, 0.16	0.01
	Placebo	1.27 ± 0.78	1.30 ± 0.79				
**Sleep Medication**	Melatonin	1.20 ± 1.06	0.60 ± 0.81	P=0.01	-0.42	-0.84, -0.007	0.08
	Placebo	1.33 ± 1.09	1.02 ± 1.05				
**Subjective sleep quality**	Melatonin	1.60 ± 1.15	1.48 ± 0.81	p =0.33	-0.20	-0.57, 0.17	0.001
	Placebo	1.73 ± 0.90	1.68 ± 0.85				
**Pelvic Pain**	Melatonin	6.83 ± 2.03	4.60 ± 2.04	**p < 0.001** ^**$**^	-1.56	-2.41, -0.71	0.18
	Placebo	6.25 ± 1.82	5.58 ± 1.56				

*Sample Size for Each Group=40

**Analysis of covariance (ANCOVA)

$ Adjusted for Baseline and Age

Our results show highly significant effects for both sleep disturbance and pelvic pain with p-values less than 0.001. These findings are robust even without formal adjustments for multiple comparisons since they fall well below the Bonferroni-adjusted significance level of p ≤ 0.006.

In terms of safety, we found no evidence for a significant side effect with about 2 months of melatonin treatment.

## Discussion

The findings of this study contribute to the growing body of evidence on the potential benefits of melatonin in managing sleep disturbances and pelvic pain associated with endometriosis. The improvements in overall sleep quality, sleep duration, sleep disturbances, sleep medication and sleep latency suggest that melatonin may be a promising adjunct therapy for infertile women with endometriosis-related sleep problems.

The relationship between sleep disturbances and chronic pain, particularly in women with endometriosis, is well-established. Patients often experience poor sleep quality, excessive daytime sleepiness, and insomnia, which correlate with increased pain severity and reduced quality of life [7]. Concerns about infertility further complicate the situation, as those facing infertility tend to experience worse sleep quality and more depressive symptoms [18]. Women with endometriosis are also more likely to use sleep medications compared to the general population [19].

Studies have shown that poor sleep not only exacerbates the perception of pelvic pain but also serves as a risk factor for its development, such as in conditions like vulvodynia [20]. Addressing these interconnected issues is crucial for improving the overall health and quality of life for women suffering from endometriosis. A pilot study using radio-wave sensing technology found that longer latency in deep sleep onset was linked to heightened pain sensitivity, although it was limited by a small sample size [21]. Moreover, the duration of pain significantly contributes to sleep disturbances, necessitating further research on this variable. Similar patterns have been observed in other chronic conditions, such as fibromyalgia [22]. Studies consistently show significant associations between sleep disturbances and various types of pelvic pain, including dysmenorrhea and dyspareunia. Addressing both pain management and sleep quality is crucial for improving the overall health and quality of life for women suffering from endometriosis [11,21]. Sleep disruption lowers pain thresholds and causes somatic symptoms, while chronic pain can lead to hyperarousal and poor sleep quality. Moderate/severe pain and its duration are associated with higher PSQI scores, indicating worse sleep quality. Chronic pelvic pain can worsen subjective sleep quality, increase sleep disturbances, and decrease sleep duration significantly. Additionally, pain duration negatively impacts subjective sleep quality and increases the use of sleep medications [23].

The potential role of melatonin in managing EACPP is increasingly supported by emerging research. Melatonin has been shown to inhibit various processes associated with endometriotic cells, including migration, invasion, epithelial-mesenchymal transition, and proliferation. This suggests that melatonin may influence the development and progression of endometriosis itself. Notably, endometriotic lesions produce estradiol and prostaglandins that promote survival, proliferation, and inflammation, which can lead to peripheral and central nerve sensitization, amplifying pain responses [24]. Melatonin’s analgesic properties may be attributed to its ability to regulate circadian rhythms. While it is well-documented that melatonin improves sleep quality, the relationship between its effects on pain and sleep remains complex. Some studies indicate that melatonin’s analgesic effects are independent of sleep quality improvements [25] The current study found that melatonin exhibited a large sized effect on chronic pelvic pain associated with endometriosis. This was evidenced by a Number Needed to Treat (NNT) of 2.03, indicating that for every approximately 3 patients treated with melatonin, one additional patient would experience a clinically meaningful reduction in pain, defined as a 2-cm decrease on the VAS, relative to those receiving a placebo. While the NNT suggests a promising therapeutic benefit, it is important to note that the observed mean difference of 1.56 may not achieve clinical significance, as the a priori hypothesis anticipated a reduction of 1.8 units on the VAS. Nonetheless, the mean difference of 1.56 is close to this threshold, indicating that melatonin could still have a meaningful impact on pain reduction for patients with endometriosis. This finding aligns with previous studies that have shown melatonin’s effectiveness in alleviating ECPP and its potential role in reducing the need for analgesic medications. This reinforces the idea that its pain-relieving properties may not solely rely on enhancing sleep quality [11] (Esmaeilzadeh et al, under review). Biologically, melatonin’s antinociceptive effects are thought to involve the activation of supraspinal sites and inhibition of spinal windup. Experimental evidence supports the idea that melatonin’s analgesic effects are mediated through interactions with opioid and gamma-aminobutyric acid (GABA) systems. Additionally, melatonin has demonstrated marked anti-inflammatory effects by inhibiting pro-inflammatory cytokine release at peripheral sites [24,25].

Despite these promising findings, the efficacy of melatonin for chronic pain management remains inconsistent across studies. For example, a clinical trial reported no significant effect of 20 mg melatonin on endometriosis pain after eight weeks [26], Conversely, other studies have highlighted beneficial effects of varying doses of melatonin across different patient populations [11,24,27]. Although melatonin is widely recognized as a sleep aid, studies have indicated that it may not always be effective or suitable at all dosages. For instance, a randomized double-blind crossover trial found that while modified-release melatonin (Circadin™) did not improve sleep disturbances at six weeks compared to placebo, it did show improvements in sleep latency and quality after three weeks, along with reductions in pain intensity [28]. Similarly, a meta-analysis indicated that while melatonin treatment reduced the severity of irritable bowel syndrome (IBS), it did not enhance sleep quality. The variability in outcomes across these studies may be attributed to differences in dosing regimens, as Circadin™ typically ranges from 2 mg to 5 mg [29]. This inconsistency underscores the need for further research to establish optimal dosing strategies and clarify the mechanisms through which melatonin exerts its effects on pain and sleep. The present study found that melatonin exhibited a large effect on overall sleep quality, as evidenced by a NNT of 1.92. This NNT value indicates that for every 2 patients treated with melatonin, one additional patient would experience a clinically meaningful improvement in sleep quality, defined as a 3-point reduction in the PSQI global score, relative to the placebo condition. This finding holds important implications for the potential utility of melatonin as an adjunct therapy for managing sleep disturbances associated with endometriosis. However, it is noteworthy that while the observed mean difference in PSQI scores between the melatonin and placebo groups was statistically significant, the magnitude of this difference (-1.7 points) did not reach the predetermined threshold for clinical meaningfulness (3-point reduction). This discrepancy between statistical and clinical significance highlights the complexities involved in interpreting sleep-related outcomes in clinical trials.

Future research should replicate these findings, explore dose-response relationships, and investigate the mechanisms underlying melatonin’s effects on sleep quality. Determining the most appropriate endpoints and clinically meaningful differences for sleep outcomes will better inform the design and interpretation of clinical trials in this field.

## Strength and limitation

The study was conducted in accordance with CONSORT guidelines, ensuring high reporting quality and transparency, which strengthens the reliability and validity of the findings. The robust, triple-blind, parallel-group, randomized controlled trial design is the gold standard for evaluating intervention efficacy, minimizing bias and enhancing internal validity. Measures to monitor treatment adherence, including regular telephone interviews, ensured the reliability of the results.

This study has several notable limitations. First, the single-center nature of the trial at the Fatemezahra Infertility Center may limit the generalizability of the findings to a broader population of endometriosis patients with sleep disturbances. Future multi-center studies involving diverse settings would help validate the results. Additionally, the study only assessed outcomes over a 2-month treatment period, and the long-term effects of melatonin on sleep quality and pelvic pain were not evaluated. Given the chronic nature of endometriosis, longer follow-up periods would be necessary to determine the sustainability of the observed improvements. However, the research team decided against a prolonged placebo-controlled study due to ethical concerns. Another limitation is the potential influence of unaccounted confounding factors, such as lifestyle habits and stress levels, which were not comprehensively assessed. The study relied solely on the self-reported PSQI questionnaire, without incorporating any objective sleep measurements, which could have provided more detailed insights into the impact of melatonin on sleep parameters. To address these limitations, the research team maintained regular communication with participants to monitor any changes in their self-management strategies. However, the inherent challenges of a placebo-controlled trial and the reliance on self-reported measures remain as limitations of this study. Future research should consider adopting a multi-center design, extending the follow-up duration, and incorporating more comprehensive assessments of potential confounding factors and objective sleep measurements.

## Conclusion

This study supports the use of melatonin to improve sleep quality in women with endometriosis-related chronic pelvic pain. The statistically significant findings highlight melatonin’s potential as a therapeutic agent for managing both pain and sleep disturbances in this population. However, the lack of clinically meaningful improvements in some outcomes suggests the need for further research to determine optimal dosing and clinical relevance. Addressing the interconnections between chronic pain, sleep, and emotional health is crucial for enhancing endometriosis management and quality of life.

## Supporting information

S1 File Study protocol. (DOCX)

S2 File CONSORT checklist. (DOCX)
