# Supplementary material for: Melatonin and sleep parameters in infertile women with endometriosis: first results from the triple-blind randomized controlled trial of administration of melatonin in chronic pelvic pain and sleep disturbance
Source: PLoS One. 2025 Apr 16;20(4):e0321635. doi: 10.1371/journal.pone.0321635 (PMC12002473; doi:10.1371/journal.pone.0321635)
Supplement: S1 File — (DOCX) [file pone.0321635.s001.docx]

**Melatonin and sleep parameters in infertile women with endometriosis: a protocol for the first results from the triple-blind randomized controlled trial of administration of melatonin in chronic pelvic pain**

**Methods and Analysis:**

**Study design**

This study is a single center; parallel group, triple blind, randomized controlled trial. This research will be approved by Institutional Review Board at Babol University of Medical Sciences. The study protocol will be prospectively registered in the website of Iranian Registry of Clinical Trials. The study protocol is based on the Standard Protocol Items: Recommendations for Interventional Trials (SPIRIT) 2013 guideline.

**Study setting:**

This trial will be performed at the Fateme Zahra infertility Center affiliated with Babol University of medical sciences and take place at endometriosis clinic. This center is one of the best equipped therapeutic infertility centers and is a referral center for infertility problems in northern Iran.

**Participants**

Eighty infertile patients diagnosed with endometriosis and sleep disorder after obtaining informed consent and meeting the eligibility criteria will be recruited in this trial. Forty patients in the experimental group taking melatonin (5 mg) and 40 participants in the comparison group and receiving placebo. The diagnosis of endometriosis is based on laparoscopy or sonography and its staging will be based on the consensus of the global endometriosis society. Patients with EACPP will receive Pittsburgh Sleep Quality Index (PSQI) questionnaire for evaluating sleep quality.

**Patient and public involvement**

Patients or the public are not involved in the design, conduct, report or dissemination of our study

**Eligibility criteria**

The eligibility of each included patients will be confirmed by the gynecologist. Subjects eligible for the study must meet all of the following **criteria at randomization:**

- **Age Range**: Participants must be between 18 and 45 years old.
- **Endometriosis Symptoms**: Individuals must report experiencing endometriosis-related chronic pelvic pain (EACPP) with a Visual Analog Scale (VAS) score of 4 or higher.
- **Sleep Disturbances**: Participants should demonstrate sleep disturbances as indicated by a Pittsburgh Sleep Quality Index (PSQI) score of 5 or greater. (Score ≥ 5).
- **Medical History**: No prior history of surgical or medical treatments related to infertility.
- **Lifestyle Factors**: Participants must not engage in smoking, alcohol consumption, or substance abuse.
- **Health Status**: Absence of systemic illnesses, including but not limited to diabetes, hypertension, and seizure disorders.

**Exclusion Criteria**

Participants will be excluded from the study if they meet any of the following conditions:

- **Medication Use**: Continuous use of sleep medications, anticoagulants, or anticonvulsants.
- **Mental Health Treatment**: Current treatment with antidepressants.
- **Stimulant Use**: Use of stimulants, hypnotics, or anti-anxiety medications.
- **Recent Mood Disorders**: A recent diagnosis (within the past 8 weeks) of mood disorders or neuropsychiatric symptoms.
- **Pregnancy/Breastfeeding**: Currently pregnant or breastfeeding.
- **Allergic Reactions**: Known hypersensitivity to melatonin.
- **Withdrawal from Study**: Participants who choose not to continue their involvement in the study.
- **Mental Health History**: A history of pharmacologically treated depression or bipolar disorder.
- **Work Schedule**: Employment that involves night shifts.

**Intervention:**

Patients will be assigned (1:1 ratio) to receive melatonin or placebo at the same time every night, approximately 2 hours before their regular bedtime. In the melatonin treatment group, patients will receive 5 mg melatonin tablet once a day for 2 months. Melatonin will be a prolonged-release formula (Jalinous Company, Melatonin 5 mg). The comparison group will receive placebo daily. Placebo treatment was identical in appearance consisting of starch (Jalinous Company).

All patients will be allowed to use analgesics (acetaminophen, ibuprofen, codeine) as needed to relieve pain, but before taking analgesic, it will be emphasized that the maximum intensity of pain should be measured and be noted the questionnaire and then use their usual medication. The number and types of analgesics used before and during the treatment will be recorded by participants and monitored monthly by the investigator. If the pain persists, patients can take acetaminophen and codeine. Patients will be asked to record the type and of amount of analgesic during treatment.

Demographic information, clinical information, ultrasound report, laparoscopy findings, and PSQI questionnaire will be completed prior to intervention. After the procedure, the questionnaire will be given to the participants along with the medicine package. The participants will be asked to complete the intervention during 2 months.

**Follow-up**

One month after intervention, participants will be monitored at the endometriosis clinic and a checklist of their pain score and possible side effects will be completed. To prevent attrition and to assess adherence to treatment, telephone interviews will be conducted every 21 days. Two months after intervention, all participants will be examined at the fertility clinic. Self-perceived PSQI questionnaire and pain VAS score will be completed again by a midwife not involved in the trial.

**Outcome measurement:**

The primary outcome measure will be the change in sleep quality after two months in patients who receiving melatonin compared to women who taking placebo. Primary outcome measure according to (PSQI) questionnaire. The PSQI consists of 19 questions to assess sleep quality in the previous month. The global PSQI score reflects the sum of the seven components (sleep duration, sleep disturbance, sleep latency, sleep efficiency, daytime dysfunction, and sleep quality) and ranges from 0–21.

[**Time Frame**: Before intervention and when two months treatments were finished in two treatment arms, sleep quality will be assessed].

We will also assess pelvic pain as a secondary endpoint of the present trial using the VAS score. The mean value of difference between baseline and 2 months later will be compared between the two groups.

Self-assessment patient’s checklist will be used to assess adherence to treatment. Reasons for dropping out and non-compliance will also be noted and reported.

**Participant timeline**

The time schedule of enrolment, interventions, and assessments based on SPIRIT 2013 template has been shown in Table1.

| **Table 1:** SPIRIT 2013 schedule of enrolment, interventions, and assessments |
| --- |

|  | **Study period** | | | | | |
| --- | --- | --- | --- | --- | --- | --- |
|  | **Enrolment** | **Allocation** | **post allocation** | | **Close- out** | |
| **Time point** | **-t_1_** | **0** | **t_1_** | **t_2_** | **t_x_** |  |
| **Enrolment** | X |  |  |  |  |  |
| Eligibility screen | X |  |  |  |  |  |
| Informed consent | X |  |  |  |  |  |
| Medical history | X |  |  |  |  |  |
| Safety index | X |  |  |  |  |  |
| Pelvic pain VAS score | X |  |  |  |  |  |
| PSQI total score  Allocation | X |  |  |  |  |  |
|  |  | X |  |  |  |  |
| **Intervention** |  |  |  |  |  |  |
| Melatonin group |  |  | X | X |  |  |
| Placebo group |  |  | X | X |  |  |
| **Assessments:** |  |  |  |  |  |  |
| sleep duration  sleep disturbance  sleep latency  sleep efficiency  daytime dysfunction  sleep quality  use of sleeping medication  PSQI total score  Pelvic pain VAS score | X |  |  |  | X |  |
|  | X |  |  |  | X |  |
|  | X |  |  |  | X |  |
|  | X |  |  |  | X |  |
|  | X |  |  |  | X |  |
|  | X |  |  |  | X |  |
|  | X |  |  |  | X |  |
|  | X |  |  |  | X |  |
|  | X |  |  |  | X |  |
| **Adverse events** |  |  | X | X | X |  |

**Sample size:**

PSQI scores will be presented as the primary outcome. The sample size was calculated based on the minimal clinically important difference (MCID) approach for sleep quality as measured by global PSQI scores for the primary outcome. In the previous study (2023, under review) we assessed the effect of melatonin on dysmenorrhea and chronic pelvic pain, by using Biberoglu and Behrman scale among 80 endometriosis patients.

We supposed means of 4.91 in melatonin and 7.91 in the placebo group ( Xu H, Zhang C et al 2020), assuming a pooled standard deviation of 3.71 and an effect size (Cohen *f*) of 0.404 in a two-tailed test with α = 0.05 and power of 0.9; The trial is powered to detect MCID=3 scores between the 2 groups at the end of the study. Allowing for a 20% drop-out rate, a sample size of 40 for each group is needed.

**“Xu H, Zhang C, Qian Y, Zou J, Li X, Liu Y, et al. Efficacy of melatonin for sleep disturbance in middle-aged primary insomnia: a double-blind, randomised clinical trial. Sleep medicine. 2020;76:113-9”**

**Recruitment**

In the run-in phase of the study a member of the research team and medical student and gynecologist will be present at endometriosis clinic in the Fatemezahra infertility center) referral center in northern Iran (4 days in a week.

The investigator will inform participants of all relevant aspects of their participation in the study and will screen all subjects for eligibility criteria at the time of enrollment. Screening of participants will continue until the target population is achieved.

All participants have an identifiable sleep disturbance by PSQI global score ≥5, followed by a confirmed diagnosis of chronic pelvic pain according to visual analog scale (VAS≥4).

**Pre-randomization data**

Demographic characteristics and a complete medical history will be obtained at the initial visit as well as through a clinical interview including age, occupation, body mass index, pelvic pain intensity, medical and drug history, endometriosis pelvic ultrasound findings, and endometriosis laparoscopy findings. After a gynecologist has determined a patient is eligible for recruitment, all eligible participants will be asked to sign a written informed consent form prior to enrollment in the study. PSQI will be completed at the start of the baseline intervention before randomization and after 2 months of treatment.

**Allocation (method & allocation concealment mechanism)**

Permuted block randomization will be used to achieve balance in the allocation of participants to intervention arms. Before initiation of the run- in phase of the study, two 80- sets of random numbers will be created by a member of the research team not involved in recruitment. Twenty blocks with size of 4 and combination of A and B will be prepared. A randomization will be made by using dedicated online software (<http://www.randomization.com>). Allocation sequence will be password-protected and only accessible to the independent investigator not involved in the study.

**Blinding**

Administration of medication type will be triple-blinded. All parties will be blinded in the
course of the random allocation sequence to avoid selection bias. Blinding of medication conditions was conducted by a pharmacist at Shahid Beheshti University of Medical Sciences. Placebo tablet, identical in size and appearance to the melatonin tablet *(*Jalinous Pharmaceutical Company) will be given to the placebo group.

**Data collection**

After check of the baseline characteristic data and eligibility criteria, the PSQI questionnaire will be completed at baseline and 2 months after enrollment. Chronic pelvic pain has been defined as the presence of intense non-cyclic pain in the pelvic area lasting for at least six months and was measured by a 10-cm VAS.

Based on previous studies and opinions of gynecologists we considered a change in sleep quality on a PSQI of more than 3 as minimal clinically important difference.

**Data Monitoring:**

A faculty member from BUMS (Dr Alijan Ahmadi Ahangar, Professor of Neurology) not involved in the trial and chosen by the vice chancellor of the university will monitor data and supervising the conduct of the study.

| Endometriosis patients referred to Fatemezahra infertility center |
| --- |

| Is patient selected for ECPP and sleep disturbance? |
| --- |

| - Confirmation of the eligibility criteria by the gynecologist  - Eligibility Screening by reproductive health researcher and by patient’s medical records  - Inviting patient to participate in the study  - Obtaining baseline characteristics  - Completed PSQI Questioner and VAS score |
| --- |

| Informed consent |
| --- |

| R**a**ndomized allocation |
| --- |

| Comparison group (n=40)  Daily for 2 months |
| --- |

| melatonin group (n=40)  Daily for 2 months |
| --- |

| Baseline assessments., Administer initial study intervention |
| --- |

| assessments of study endpoints and safety |
| --- |

| Participants will be prescribed medication during the first visit where recruitment of the study occurs. Three weeks after the treatment, a telephone interview will be conducted to clarify side effects and to remind and monitor their medications. If participants withdrawn for any reason, they will be asked to complete an early termination assessment. They should be seen in the clinic as soon as possible following discontinuation of the study drug. |  |
| --- | --- |

**Statistics and data-analysis**

Baseline characteristics will be compared between melatonin and placebo groups by using independent *t*-test for the normally distributed continuous variables and Chi-squared tests for categorical variables. If the assumptions for normality are violated then non-parametric techniques will be applied.

The primary outcome variable, change in PSQI score, will be calculated by subtracting PSQI scores at follow-up from PSQI scores at baseline. For the primary outcome measurement, the mean difference of parameters between the melatonin and placebo groups will be analyzed using One-way analysis of covariance (ANCOVA) with between-group differences over time (pre-intervention to post-intervention). Effect sizes were expressed in standardized mean differences (Cohen’s d), where a value of 0.2, 0.5, and 0.8 reflects a small, medium, and large effect size, respectively (Cohen, 1988). Statistical analysis will be performed using the Statistics Package for Social Sciences software (version 22, SPSS, Chicago, IL, USA) and Stata 17 (Stata Corp, College Station, TX, USA) according to the intention-to-treat (ITT) approach, with two-sided uncertainty and type I error 0.05.

**Trial status**

The protocol version number: Latest Version. Recruitment will be started on 16 July 2023.

**Abbreviations:**

EACPP: endometriosis-associated chronic pelvic pain; PSQI: Pittsburgh Sleep Quality Index; VAS: visual analog scale; BDNF: brain-derived neurotropic factor; MCID: minimal clinically important difference;

## Declarations

## Ethics approval and consent to participate

The research project has received the confirmation of the Institution Ethics Committee (Babol University of Medical Sciences) with the number MUBABOL.HRI.REC.1402.041 Dated 03/07/2023.

The consent form of our patients will be taken and will be available.Authors contributions

## PM the Chief Investigator; she conceived the study, led the proposal and protocol development. SE, and ZB contributed to study design and to development of the proposal. DM was the lead trial methodologist. HGH will contribute to the interpretation of the data; FH and MGA will draft the manuscript. All authors read and approved the final manuscript. FH and SE are co-first author.

## Funding

This research is funded by vice chancellor of Research and technology of Babol University of medical sciences. Both melatonin and placebo produced by Jalinous Pharmaceuticals Company.

The sponsors had no such involvement.

Dissemination policy

Study findings will be communicated to participants, endometriosis specialist, and the public via reporting the results at medical society meetings. Most importantly, we plan to publish the study results in peer-reviewed journals
